# Discussion of Dr. Toepel’s Paper

**Published:** 1907-05

**Authors:** 


### DISCUSSION OF DR. TOEPEL'S PAPER.

---

DR. R. R. KIME urged that the medical profession generally devote more study and time to the subject of exercise and that medical colleges give better courses of instruction in this branch of medicine.

DR. MICHAEL HOKE mentioned the importance of massage and proper exercise in the after-treatment of sprains, and thought many of the distorted and deformed joints could have been avoided if the proper treatment had been received along this line. Dr. Hoke complimented very highly the excellent work done by Dr. Toepel.

DR. W. A. CROWE spoke of the history of massage and exercise, especially as regards pelvic disorders and neurotic conditions. He also appreciated the work of Dr. Toepel and believed that patients needing after-treatment of this kind should always be advised to go to a skilled and scientific physician with special knowledge on this line, rather than to allow them to fall into the hands of quacks and osteopaths later and bring reproach upon the medical profession.

DR. M. A. PURSE related the history of a patient illustrating the difficulty sometimes experienced by physicians in pursuading those needing treatment to go to the right party, as they frequently prefer taking the advice of a friend or assume charge of their own case and seek the advice of a much-advertised quack. He regarded very highly the work of Dr. Toepel.

DR. ARCHIBALD SMITH also found it difficult to have these patients carry out his advice or submit to his treatment, as they think the osteopath has more experience and knowledge of this form of treatment.

DR. TOEPEL (closing) showed that there was absolutely nothing new in osteopathy, but that they had really stolen the Swedish Medical Massage and Gymnastics and claimed them as their own because physicians did not recognize the value of this treatment until too late. In 1809, Peter Henry Ling, of Stockholm, Sweden, introduced physical exercise based on physiology and gradually developed what is now known as the Swedish Medical Gymnastics, which comprises the passive movements commonly known as massage, and the active and resistive exercises. This system is extensively used all over Europe by every intelligent physician, and for this reason there are no osteopaths or very few of them to be seen there. If the physicians of this country gave their patients suitable treatment with exercise and massage when indicated, the majority of them would be prevented from ever reaching the osteopaths.

